# Supplementary material for: Causal Relationship between Aging and Anorexia Nervosa: A White-Matter-Microstructure-Mediated Mendelian Randomization Analysis
Source: Biomedicines. 2024 Aug 16;12(8):1874. doi: 10.3390/biomedicines12081874 (PMC11351342; doi:10.3390/biomedicines12081874)
Supplement: Supplementary file 1 [file biomedicines-12-01874-s001.zip › Supplementary Figure/Figure S4/Singe SNP plot of MR analysis for TL to ubm-b-1795.pdf]

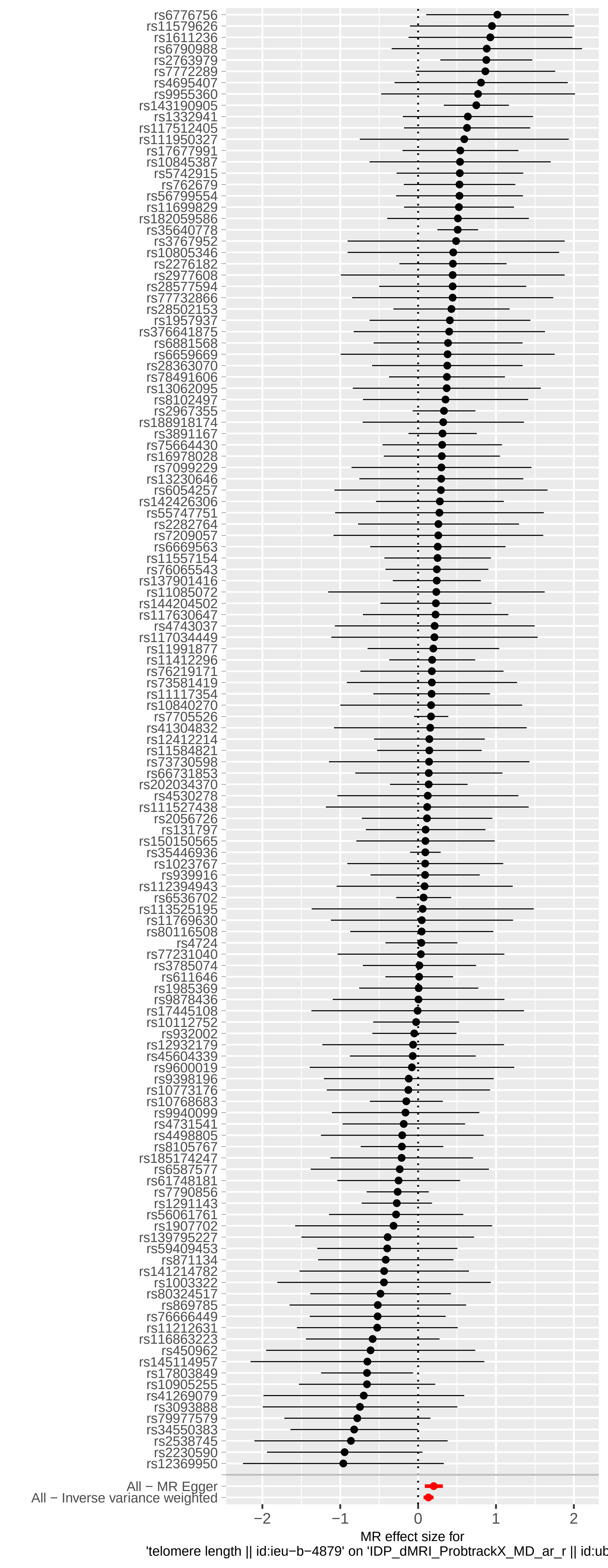

rs6776756
rs11579626
rs1611236
rs6790988
rs2763979
rs7772289
rs4695407
rs9955360
rs143190905
rs1332941
rs117512405
rs111950327
rs17677991
rs10845387
rs5742915
rs762679
rs56799554
rs11699829
rs182059586
rs35640778
rs3767952
rs10805346
rs2276182
rs2977608
rs28577594
rs77732866
rs28502153
rs1957937
rs376641875
rs6881568
rs6659669
rs28363070
rs78491606
rs13062095
rs8102497
rs2967355
rs188918174
rs3891167
rs75664430
rs16978028
rs7099229
rs13230646
rs6054257
rs142426306
rs55747751
rs2282764
rs7209057
rs6669563
rs11557154
rs76065543
rs137901416
rs11085072
rs144204502
rs117630647
rs4743037
rs117034449
rs11991877
rs11412296
rs76219171
rs73581419
rs11117354
rs10840270
rs7705526
rs41304832
rs12412214
rs11584821
rs73730598
rs66731853
rs202034370
rs4530278
rs111527438
rs2056726
rs131797
rs150150565
rs35446936
rs1023767
rs939916
rs112394943
rs6536702
rs113525195
rs11769630
rs80116508
rs4724
rs77231040
rs3785074
rs611646
rs1985369
rs9878436
rs17445108
rs10112752
rs932002
rs12932179
rs45604339
rs9600019
rs9398196
rs10773176
rs10768683
rs9940099
rs4731541
rs4498805
rs8105767
rs185174247
rs6587577
rs61748181
rs7790856
rs1291143
rs56061761
rs1907702
rs139795227
rs59409453
rs871134
rs141214782
rs1003322
rs80324517
rs869785
rs76666449
rs11212631
rs116863223
rs450962
rs145114957
rs17803849
rs10905255
rs41269079
rs3093888
rs79977579
rs34550383
rs2538745
rs2230590
rs12369950
All − MR Egger
All − Inverse variance weighted
−2
−1
0
1
2
MR effect size for
'telomere length || id:ieu−b−4879' on 'IDP_dMRI_ProbtrackX_MD_ar_r || id:ub
